# Supplementary material for: Genomic epidemiology demonstrates spatially clustered, local transmission of Plasmodium falciparum in forest-going populations in southern Lao PDR
Source: PLoS Pathog. 2024 Sep 23;20(9):e1012194. doi: 10.1371/journal.ppat.1012194 (PMC11449315; doi:10.1371/journal.ppat.1012194)
Supplement: S1 Fig — (DOCX) [file ppat.1012194.s001.docx]

**
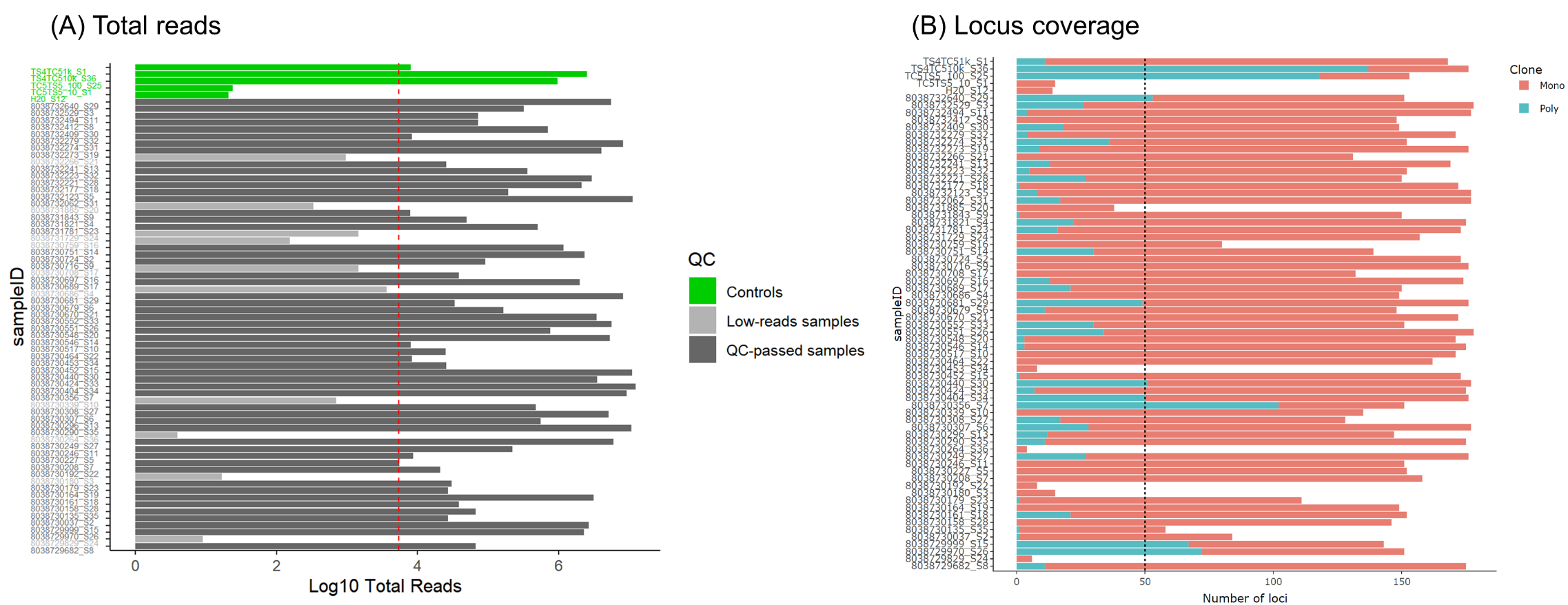
**

**S1 Fig. Quality control (QC) of sequenced positive samples.** A total of 65 positive cases were subjected to sequencing and recovering 180 diverse loci. Out of these, 53 samples (82%) successfully passed the quality control using two filters: (A) Total read counts > total number of loci * 30, (B) Locus coverage > 50 out of 180 (Note: "mono" denotes a locus with a single allele, while "poly" indicates a locus with more than one allele). The dashed lines in both plots represent the filtering thresholds. Only the samples that passed both thresholds were retained for downstream analysis.
