# Supplementary material for: Genomic epidemiology demonstrates spatially clustered, local transmission of Plasmodium falciparum in forest-going populations in southern Lao PDR
Source: PLoS Pathog. 2024 Sep 23;20(9):e1012194. doi: 10.1371/journal.ppat.1012194 (PMC11449315; doi:10.1371/journal.ppat.1012194)
Supplement: S2 Fig — (DOCX) [file ppat.1012194.s002.docx]

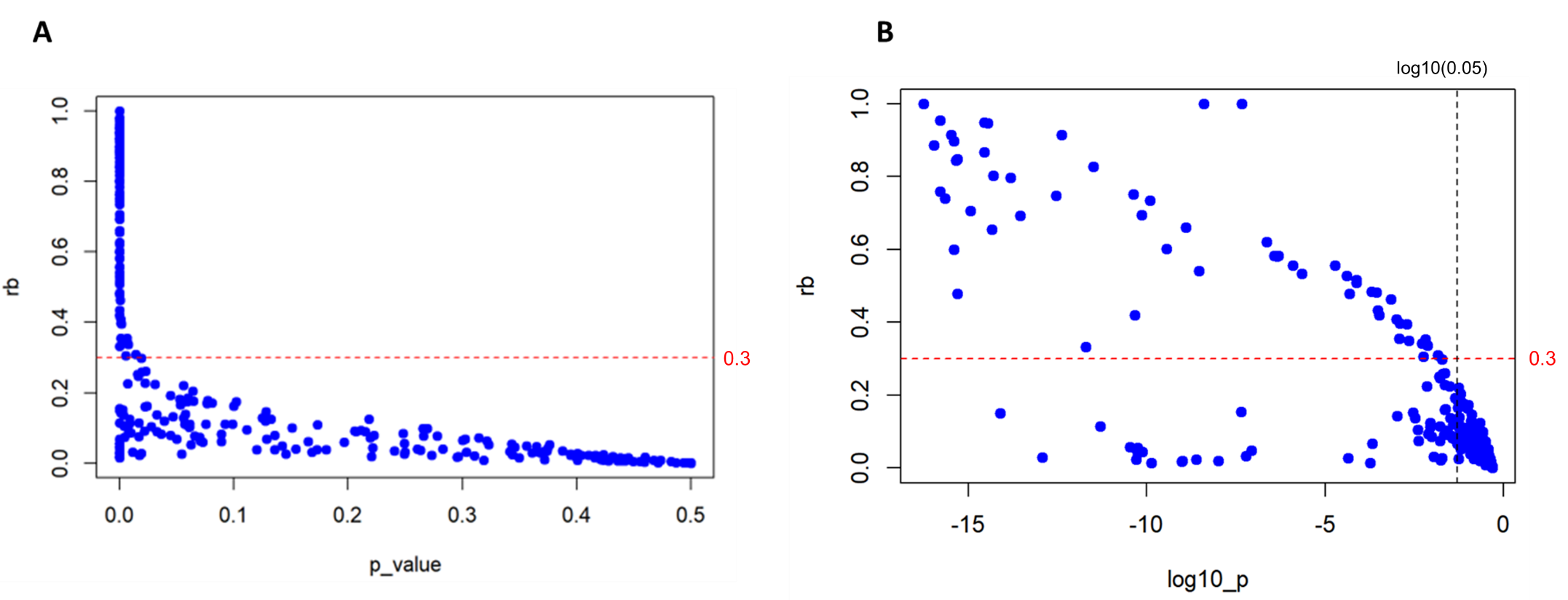


**S2 Fig.** **Distribution of** $\hat{\boldsymbol{rb}}$ **with corresponding p-value (A) and log10-transformed p-value (B).**

The p-values are divided by two for one-sided tests. The variation in p-values for $\hat{rb}$ ≤ 0.3 (red dashed line) increased, leading to more non-significant pairs. Therefore, a minimum $\hat{rb}$cutoff of 0.3 (red dashed line) was used to define related pairs with a significance level of 0.05 (black dashed line), where all the pairs had a $\hat{rb}$ ≥ 0.3.
