# Supplementary material for: Genomic epidemiology demonstrates spatially clustered, local transmission of Plasmodium falciparum in forest-going populations in southern Lao PDR
Source: PLoS Pathog. 2024 Sep 23;20(9):e1012194. doi: 10.1371/journal.ppat.1012194 (PMC11449315; doi:10.1371/journal.ppat.1012194)
Supplement: S3 Fig — (DOCX) [file ppat.1012194.s003.docx]

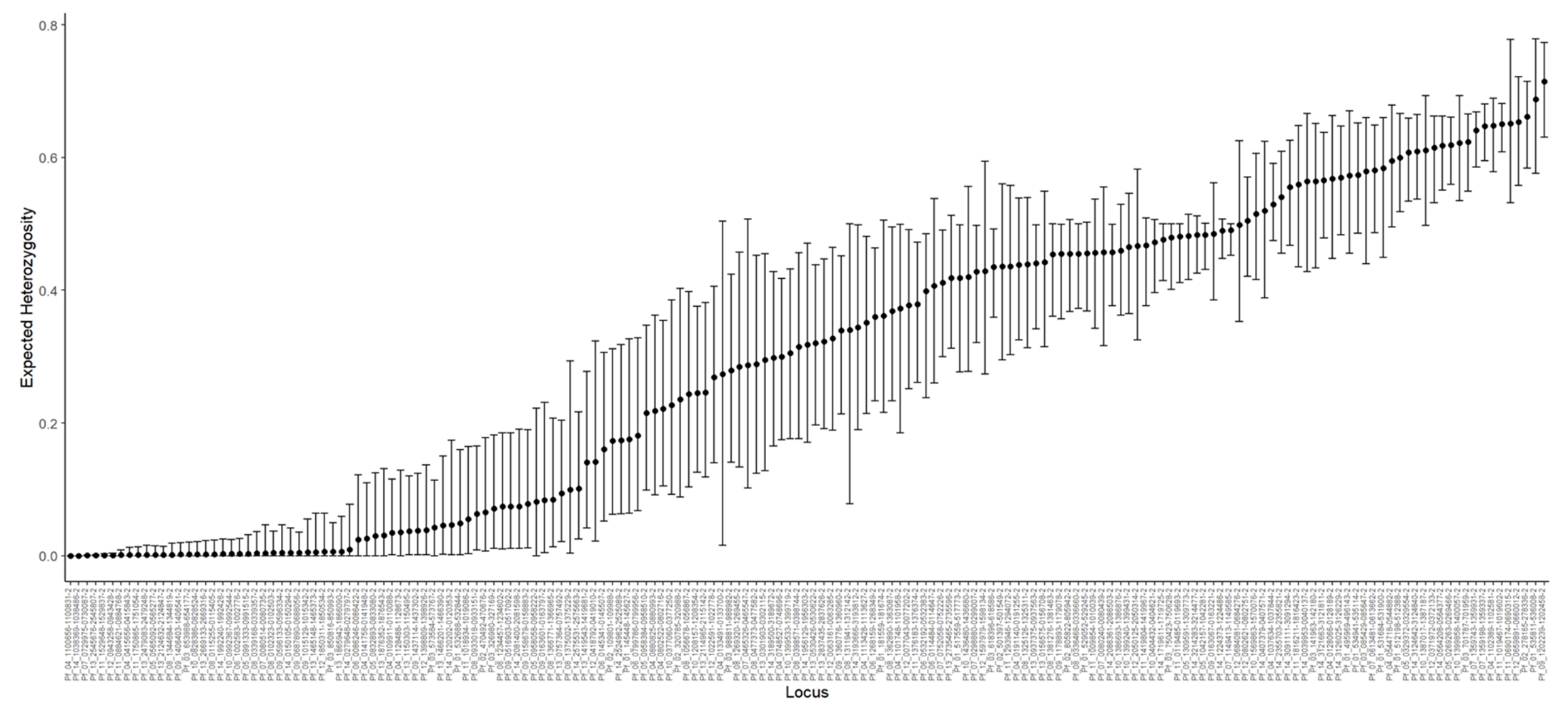


**S3 Fig. Expected heterozygosity in 180 highly polymorphic loci.** The dots indicate the mean estimates, and the bars show 95% credible interval.
