# Supplementary material for: Genomic epidemiology demonstrates spatially clustered, local transmission of Plasmodium falciparum in forest-going populations in southern Lao PDR
Source: PLoS Pathog. 2024 Sep 23;20(9):e1012194. doi: 10.1371/journal.ppat.1012194 (PMC11449315; doi:10.1371/journal.ppat.1012194)
Supplement: S4 Fig — (DOCX) [file ppat.1012194.s004.docx]

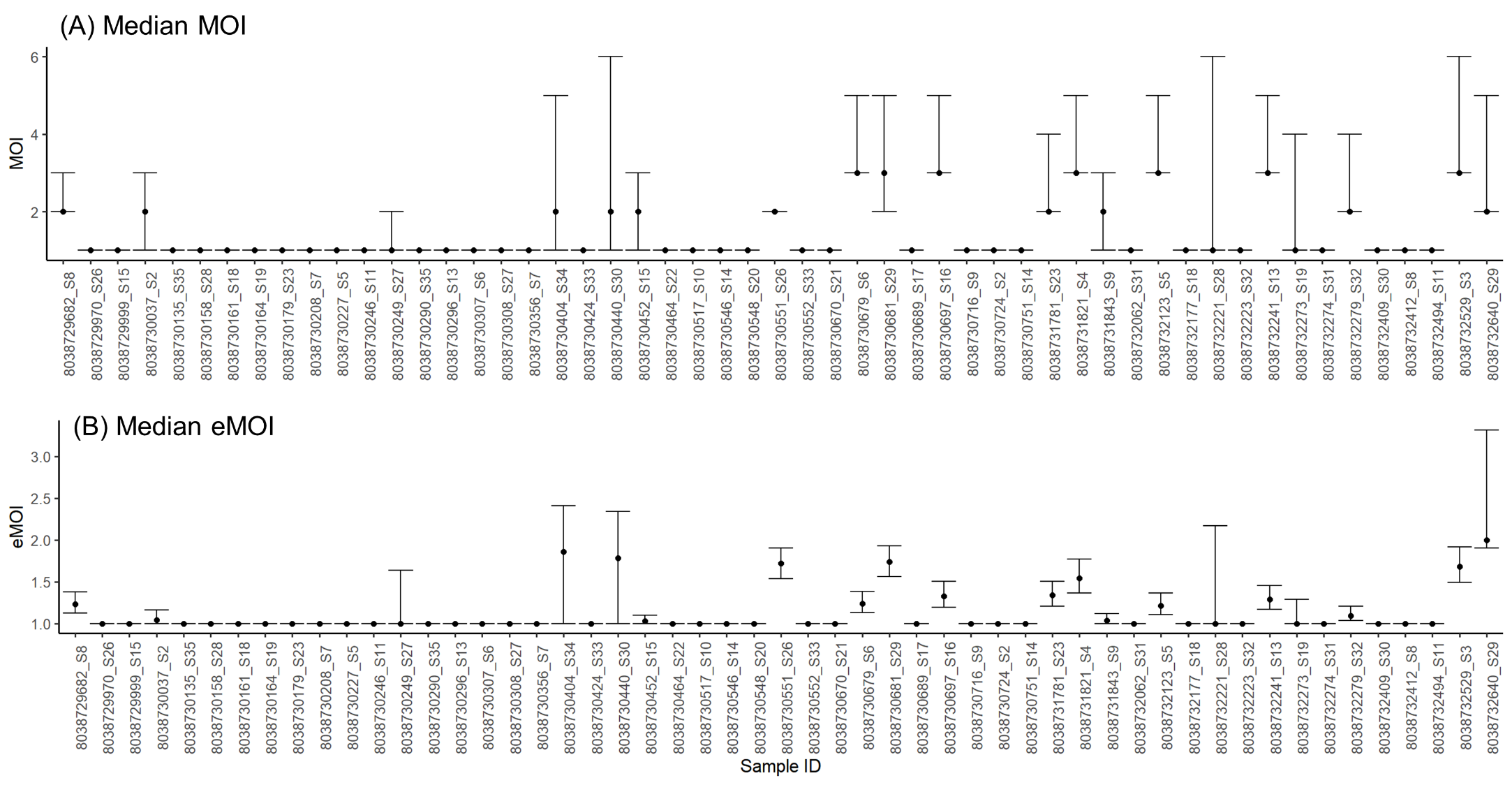


**S4 Fig. Median MOI and eMOI in each sample.** The upper plot displays the median MOI, while the lower plot represents the median effective MOI. The bars show 95% credible interval of the posterior distribution of MOI and eMOI, and the dots indicate the median.
