# Supplementary figures and images for: Genomic epidemiology demonstrates spatially clustered, local transmission of Plasmodium falciparum in forest-going populations in southern Lao PDR
Source: PLoS Pathog. 2024 Sep 23;20(9):e1012194. doi: 10.1371/journal.ppat.1012194 (PMC11449315; doi:10.1371/journal.ppat.1012194)

**
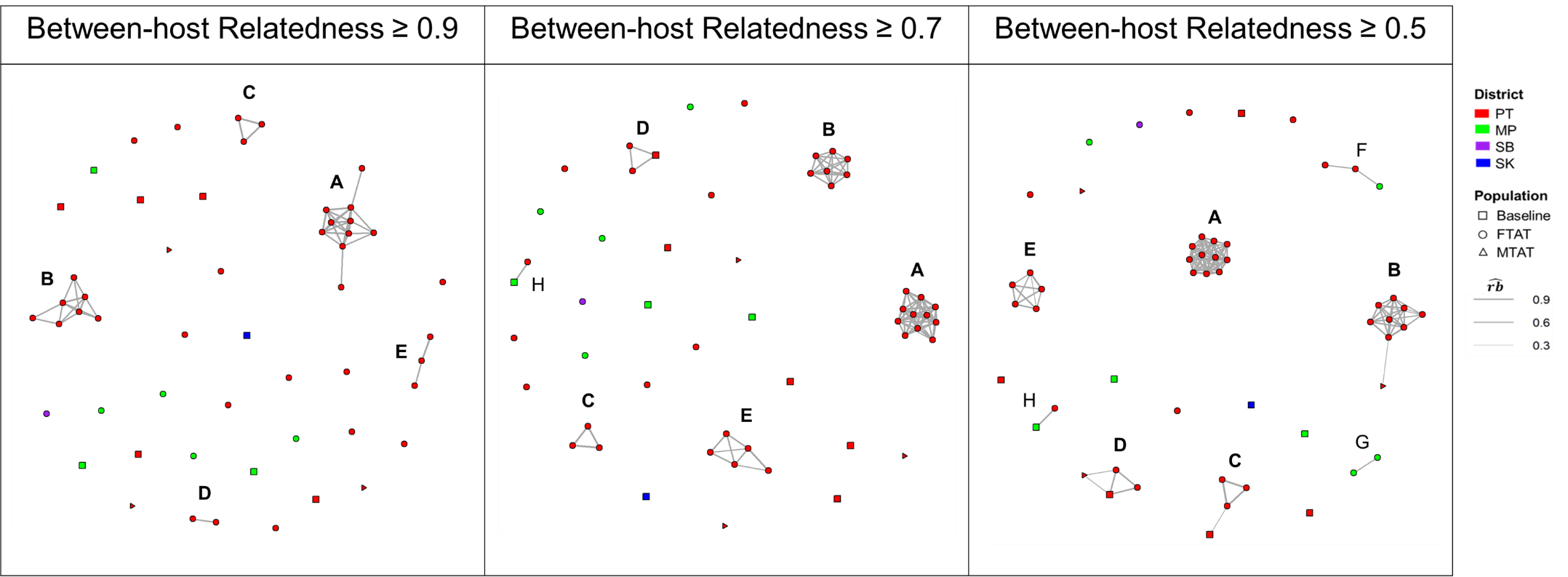
**

**S5 Fig. Clustering network with different between-host relatedness cutoffs.**

Supplement: S5 Fig — (DOCX) [file ppat.1012194.s005.docx]
