# Supplementary material for: Genomic epidemiology demonstrates spatially clustered, local transmission of Plasmodium falciparum in forest-going populations in southern Lao PDR
Source: PLoS Pathog. 2024 Sep 23;20(9):e1012194. doi: 10.1371/journal.ppat.1012194 (PMC11449315; doi:10.1371/journal.ppat.1012194)
Supplement: S6 Fig — (DOCX) [file ppat.1012194.s006.docx]

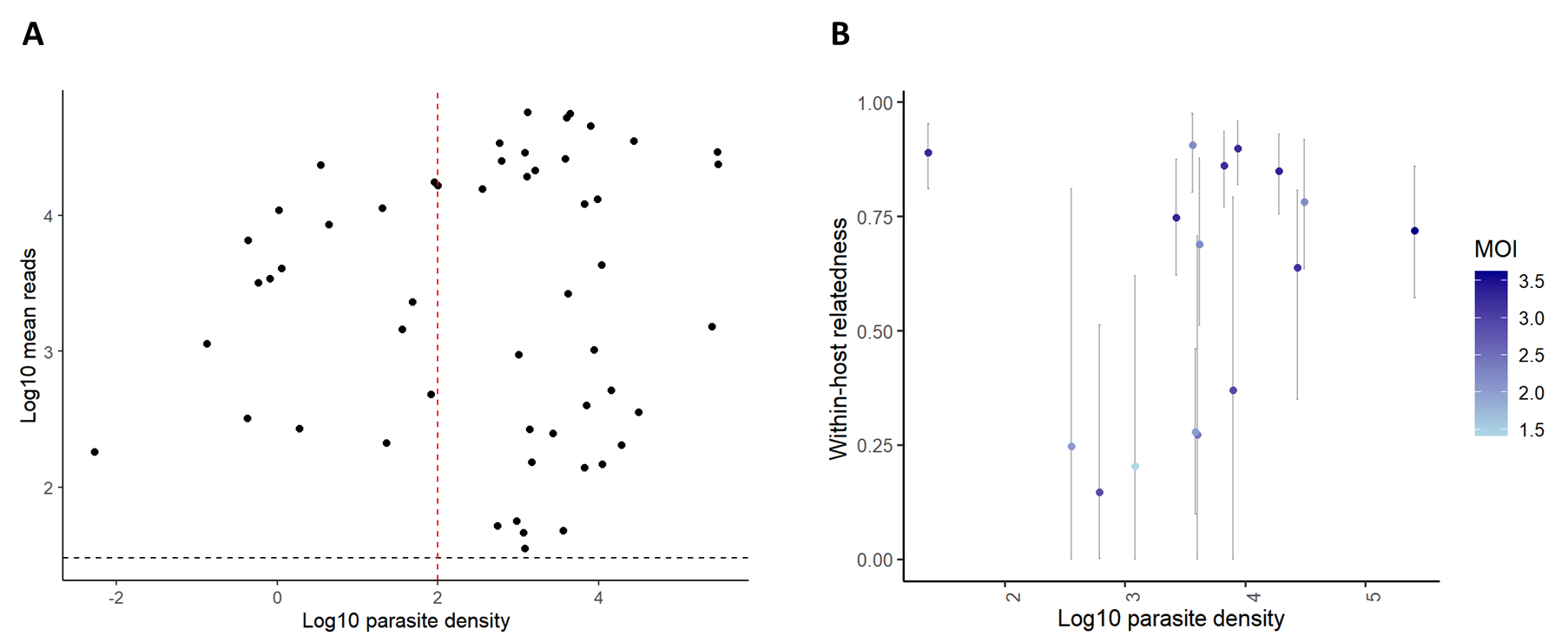


**S6 Fig. Relationship between parasite density, sequenced reads (A), and within-host relatedness (B).**

Parasite density was measured using the *varATS* qPCR. (A) In low-density samples with <100 parasites/uL (red dashed line), read counts performed similarly to high-density samples, with an average of at least 30 reads per amplicon (black dashed line), passing our quality control criterion. (B) Parasite density and within-host relatedness showed no significant correlation (Pearson’s p-value = 0.37). However, the confidence interval for within-host relatedness can be larger when the estimated within-host relatedness is lower and/or MOI is lower. This suggests that the uncertainty of within-host relatedness can be influenced by MOI estimates, rather than parasite density.
