# Supplementary material for: Genomic epidemiology demonstrates spatially clustered, local transmission of Plasmodium falciparum in forest-going populations in southern Lao PDR
Source: PLoS Pathog. 2024 Sep 23;20(9):e1012194. doi: 10.1371/journal.ppat.1012194 (PMC11449315; doi:10.1371/journal.ppat.1012194)
Supplement: S1 Table — (DOCX) [file ppat.1012194.s007.docx]

**S1 Table. Number of positive and sequenced samples.**

| **Survey (Time)** | **No. of collection** | **No. of PCR Pf-positive samples** | **No. of usable sequenced samples** |
| --- | --- | --- | --- |
| Baseline (Nov. to Dec. 2017) | 5,749 | 14 | 9 |
| FTAT (Mar. to Nov. 2018) | 2,904 | 48 | 41 |
| MTAT (Jun. to Jul. 2018) | 18,144 | 3 | 3 |
| Endline (Oct. to Nov. 2018) | 7,870 | 0 | 0 |
| **Total (%)** | **34,667** | **65 (0.19%)** | **53 (0.15%)** |
