# Supplementary material for: Genomic epidemiology demonstrates spatially clustered, local transmission of Plasmodium falciparum in forest-going populations in southern Lao PDR
Source: PLoS Pathog. 2024 Sep 23;20(9):e1012194. doi: 10.1371/journal.ppat.1012194 (PMC11449315; doi:10.1371/journal.ppat.1012194)
Supplement: S2 Table — (DOCX) [file ppat.1012194.s008.docx]

**S2 Table. Pairwise relatedness and proportion of significant pairs.**

| **Relatedness** | **Significant pairs  (p < 0.05)** | **Total pairs** | **% of significantly related pairs** |
| --- | --- | --- | --- |
| 0.0 – 0.1 | 27 | 1197 | 2 |
| 0.1 – 0.2 | 17 | 45 | 38 |
| 0.2 – 0.3 | 9 | 11 | 82 |
| 0.3 – 0.4 | 10 | 10 | 100 |
| 0.4 – 0.5 | 9 | 9 | 100 |
| 0.5 – 0.6 | 12 | 12 | 100 |
| 0.6 – 0.7 | 7 | 7 | 100 |
| 0.7 – 0.8 | 12 | 12 | 100 |
| 0.8 – 0.9 | 28 | 28 | 100 |
| 0.9 – 1.0 | 47 | 47 | 100 |

There is a total of 1,378 (53^2^ – 53 / 2) distinct sample pairs. We applied a significance level at p < 0.05.
