# Supplementary material for: Genomic epidemiology demonstrates spatially clustered, local transmission of Plasmodium falciparum in forest-going populations in southern Lao PDR
Source: PLoS Pathog. 2024 Sep 23;20(9):e1012194. doi: 10.1371/journal.ppat.1012194 (PMC11449315; doi:10.1371/journal.ppat.1012194)
Supplement: S3 Appendix — (DOCX) [file ppat.1012194.s011.docx]

**Appendix file 4 – Cluster dendrogram with parasites from other countries (A) and drug resistance analysis (B)**

**(A) Cluster dendrogram between study parasites and** **contemporaneous parasites from other countries**

An additional analysis was conducted to assess the genetic relatedness between the study parasites and other contemporaneous parasites circulating in Laos (national), Vietnam (a neighboring country), and Ghana (a distant country), using WGS data from 50 samples per country available from the MalariaGEN project [1]. Comparison between the study samples and WGS data was performed by first running local haplotype assembly with *PathWeaver* [2] on the regions corresponding to the amplicon panel used in the study. Relatedness between samples was calculated by using *Dcifer* [3].

The cluster dendrogram below showed that aside from the outgroup of Ghana samples, 66% (35/53) of our study samples (light blue dots) were mainly clustered with other Laos samples (dark blue dots), while the remaining 34% (18/53) of the study samples were closer to a mix of Laos and Vietnam samples. The majority of our samples tended to cluster together, with only 2 non-clustered samples being distinctive haplotypes. This indicates that the majority of our study samples may be locally acquired and clustered, supporting our inference in lines 241-243 that 81% of our captured cases are related to at least one other infection and are likely to be locally clustered, rather than imported.


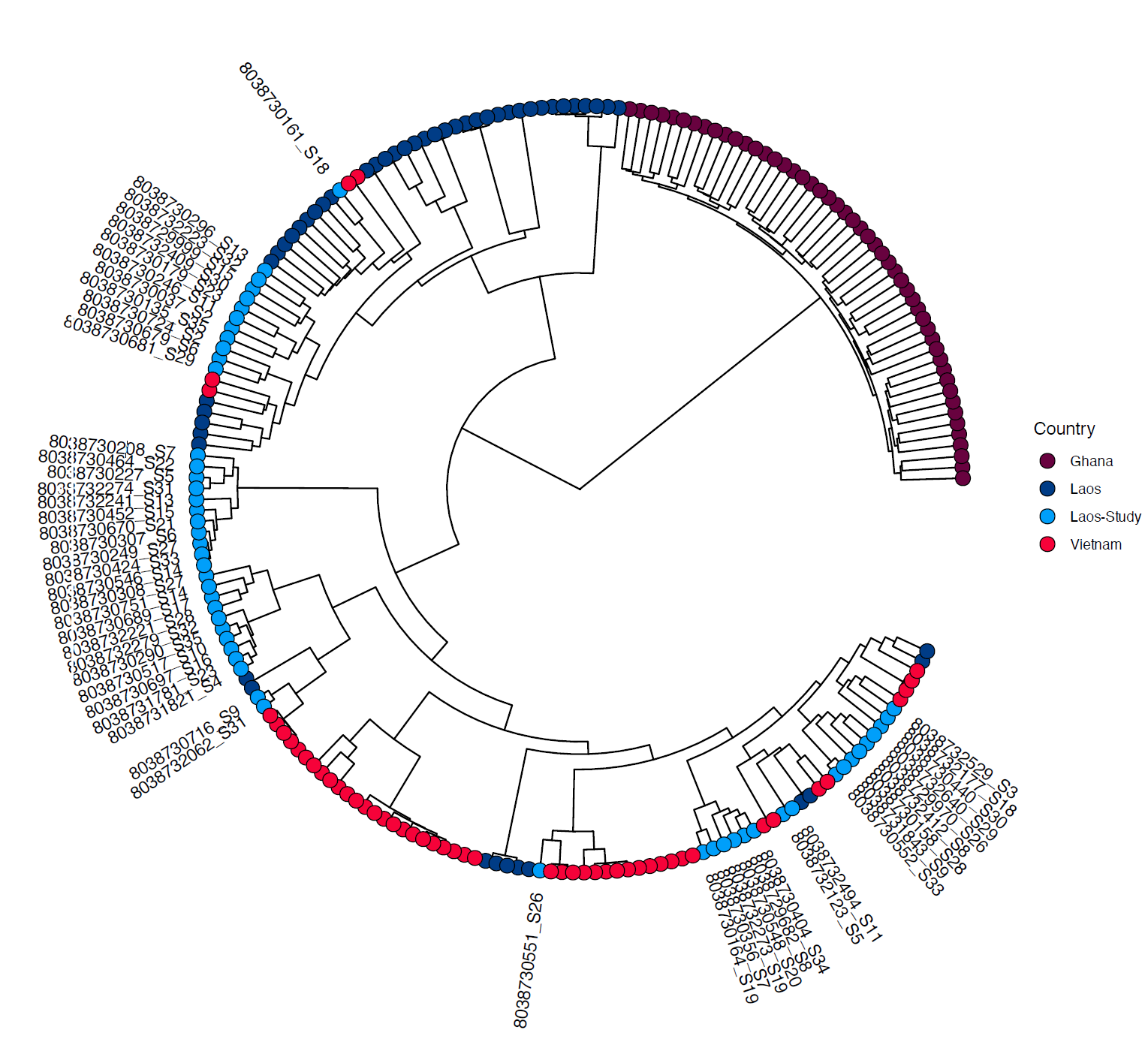


**(B) Drug resistance analysis of 53 Pf-positive cases**

In this amplicon sequencing panel, we aimed to amplify a total of 204 amplicons, including 7 drug-resistance loci in *dhfr*, *dhps*, *mdr1*, *mdr2*, and *k13*. This particular primer set was designed with a focus on genetic diversity in Southeast Asia, and the drug resistance markers were not as robust. We failed to retrieve sequences from 2 amplicons across samples, *dhps* and *k13*. For the remaining 5 amplicons (2 in *dhfr*, 2 in *mdr1*, and 1 in *mdr2*), 1 to 6 microhaplotypes were identified in each amplicon, with a dominant haplotype accounting for 81-100% of the population allele frequency. Upon inspecting the dominant haplotypes in known drug-resistance sites of *dhfr*, *mdr1*, and *mdr2* across our sequenced cases, we identified the following mutants associated with decreased sensitivity: 51I (100%), 59R (100%), 108N (100%), and 164L (81%) in the *dhfr* gene for decreased sensitivity to sulfadoxine-pyrimethamine; N86 (96%) and 184F (88%) in *mdr1* for decreased sensitivity to lumefantrine; and 484I (81%) in *mdr2* as possible background mutation to artemisinin [4].

References

1. Ahouidi A, Ali M, Almagro-Garcia J, Amambua-Ngwa A, Amaratunga C, Amato R, et al. An open dataset of Plasmodium falciparum genome variation in 7,000 worldwide samples. Wellcome open research. 2021;6:42. Epub 2021/07/30. doi: 10.12688/wellcomeopenres.16168.2. PubMed PMID: 33824913; PubMed Central PMCID: PMCPMC8008441.

2. Hathaway N. A suite of computational tools to interrogate sequence data with local haplotype analysis within complex Plasmodium infections and other microbial mixtures. 2018.

3. Gerlovina I, Gerlovin B, Rodríguez-Barraquer I, Greenhouse B. Dcifer: an IBD-based method to calculate genetic distance between polyclonal infections. <https://eppicenter.github.io/dcifer/>. Genetics. 2022;222(2). doi: 10.1093/genetics/iyac126.

4. Ndong Ngomo JM, Mawili-Mboumba DP, M'Bondoukwé NP, Ditombi BM, Koumba Lengongo JV, Batchy Ognagosso FB, et al. Drug Resistance Molecular Markers of Plasmodium falciparum and Severity of Malaria in Febrile Children in the Sentinel Site for Malaria Surveillance of Melen in Gabon: Additional Data from the Plasmodium Diversity Network African Network. Tropical medicine and infectious disease. 2023;8(4). Epub 2023/04/27. doi: 10.3390/tropicalmed8040184. PubMed PMID: 37104310; PubMed Central PMCID: PMCPMC10147079.
